# CT-based clinical-radiomics model to predict progression and drive clinical applicability in locally advanced head and neck cancer

**DOI:** 10.1007/s00330-024-11301-6

**Published:** 2024-12-20

**Authors:** Gema Bruixola, Delfina Dualde-Beltrán, Ana Jimenez-Pastor, Anna Nogué, Fuensanta Bellvís, Almudena Fuster-Matanzo, Clara Alfaro-Cervelló, Nuria Grimalt, Nader Salhab-Ibáñez, Vicente Escorihuela, María Eugenia Iglesias, María Maroñas, Ángel Alberich-Bayarri, Andrés Cervantes, Noelia Tarazona

**Affiliations:** 1 Medical Oncology Department, Hospital Clinico Universitario de Valencia—INCLIVA Biomedical Research Institute, University of Valencia, Valencia, Spain; 2 Radiology Department, Hospital Clinico Universitario de Valencia, University of Valencia, Valencia, Spain; 3 Quibim—Quantitative Imaging Biomarkers in Medicine, Valencia, Spain; 4 Pathology Department, Hospital Clinico Universitario de Valencia—INCLIVA Instituto de Investigación Sanitaria, University of Valencia, Valencia, Spain; 5 Otorhinolaryngology Department, Hospital Clinico Universitario de Valencia, Valencia, Spain; 6 Oral and Maxillary Surgery Department, Hospital Clinico Universitario de Valencia, Valencia, Spain; 7 Radiation Oncology Department, Hospital Clinico Universitario de Valencia, Valencia, Spain; 8 Instituto de Salud Carlos III, CIBERONC, Madrid, Spain

**Keywords:** Radiomics, Imaging biomarkers, CT, Head and neck cancer, PFS

## Abstract

**Background:**

Definitive chemoradiation is the primary treatment for locally advanced head and neck carcinoma (LAHNSCC). Optimising outcome predictions requires validated biomarkers, since TNM8 and HPV could have limitations. Radiomics may enhance risk stratification.

**Methods:**

This single-centre observational study collected clinical data and baseline CT scans from 171 LAHNSCC patients treated with chemoradiation. The dataset was divided into training (80%) and test (20%) sets, with a 5-fold cross-validation on the training set. Researchers extracted 108 radiomics features from each primary tumour and applied survival analysis and classification models to predict progression-free survival (PFS) and 5-year progression, respectively. Performance was evaluated using inverse probability of censoring weights and *c*-index for the PFS model and AUC, sensitivity, specificity, and accuracy for the 5-year progression model. Feature importance was measured by the SHapley Additive exPlanations (SHAP) method and patient stratification was assessed through Kaplan–Meier curves.

**Results:**

The final dataset included 171 LAHNSCC patients, with 53% experiencing disease progression at 5 years. The random survival forest model best predicted PFS, with an AUC of 0.64 and CI of 0.66 on the test set, highlighting 4 radiomics features and TNM8 as significant contributors. It successfully stratified patients into low and high-risk groups (log-rank *p* < 0.005). The extreme gradient boosting model most effectively predicted a 5-year progression, incorporating 12 radiomics features and four clinical variables, achieving an AUC of 0.74, sensitivity of 0.53, specificity of 0.81, and accuracy of 0.66 on the test set.

**Conclusion:**

The combined clinical-radiomics model improved the standard TNM8 and clinical variables in predicting 5-year progression though further validation is necessary.

**Key Points:**

***Question*** *There is an unmet need for non-invasive biomarkers to guide treatment in locally advanced head and neck cancer*.

***Findings*** *Clinical data (TNM8 staging, primary tumour site, age, and smoking) plus radiomics improved 5-year progression prediction compared with the clinical comprehensive model or TNM staging alone*.

***Clinical relevance*** *SHAP simplifies complex machine learning radiomics models for clinicians by using easy-to-understand graphical representations, promoting explainability*.

## Introduction

Head and neck squamous cell carcinoma (HNSCC), is the seventh most prevalent cancer worldwide [1]. Major risk factors are tobacco, alcohol [2], and human papillomavirus (HPV) infection [3, 4].

Over 60% of new HNSCC cases are locally advanced head and neck cancer (LAHNSCC). Therapeutic decisions are influenced by location, TNM stage, patient age, and comorbidities [5, 6]. The standard definitive treatment is definitive high-dose cisplatin with concurrent radiotherapy [7] or radiotherapy with cetuximab in unfit patients [7, 8]. Despite optimal therapy, recurrence rates are significant.

The 8th edition of the cancer staging manual [9] refined the prognostic stratification of HNSCC, but it remains suboptimal [10]. Gene expression also impacts HNSCC prognosis [11] yet no molecular classification has been implemented in clinical practice [12].

CT is commonly used for staging and monitoring HNSCC, providing detailed cross-sectional images to pinpoint tumour location, size, lymph node involvement, and adjacent structure evaluation. Visual interpretation of computed tomography (CT) scans poses challenges, especially in distinguishing early inflammatory/fibrotic changes from active disease post-radical treatment [13, 14].

Radiomics applies computational analysis of medical images to extract detailed, quantitative and higher-order, subvisual aiding clinical decision-making through advanced algorithms [15]. In head and neck cancer, several radiomic models have been developed to improve diagnosis, prognostic estimation, and toxicity prediction, focusing on variables like histological grade, ENE+, PD-L1 expression, HPV status, and treatment-related toxicities such as trismus and xerostomia. Additionally, some radiogenomic models have been proposed for predicting molecular subtypes and mutation status. However, these models face significant limitations due to a lack of external validation, reproducibility, and the high heterogeneity of study populations in terms of cancer stages and treatments, making it challenging to translate these findings into clinical practice. As a result, none of these models have been successfully implemented [16]. Furthermore, there is no standardised, universally accepted panel of radiomics variables validated for clinical use. Moreover, the opaque nature of machine learning (ML) models makes patient-specific predictions difficult to explain [17].

To address the interpretability issue, the Shapley additive explanations (SHAP) method was introduced [18]. SHAP assigns positive or negative values to features to show the direction of their influence, with higher values indicating greater importance.

We hypothesised that a combined model, incorporating clinical and biological variables and radiomics features, might outperform the current standard (tumour-node-metastases eight editions (TNM8) and HPV)) in stratifying LAHNSCC patients into progression risk groups. We aimed to build a CT-based radiomics model with clinic-biological data integrated into a multidimensional signature for progression-free survival [PFS] prediction with SHAP values to provide an intuitive explanation of the decision-making process.

## Materials and methods

### Study design and patient population

This observational study included patients diagnosed with LAHNSCC eligible for definitive concurrent radiation with either cisplatin or cetuximab from 2016 to 2023 at Hospital Clínico Universitario de Valencia (HCUV). Patient data were gathered from both retrospective (January 2016–January 2017) and prospective cases (February 2017–September 2023).

The treatment decision was taken by the Multidisciplinary Head and Neck Tumours Committee of the HCUV. Inclusion and exclusion criteria are detailed in the Supplementary Material.

TNM8 American Joint Committee on Cancer staging system [9]. HPV status was determined through p16 staining and HPV deoxyribonucleic acid genotype analysis [4].

This work adhered to the guidelines outlined in the transparent reporting of a multivariable prediction model for individual prognosis or diagnosis statements [19].

This study was approved by the HCUV Institutional Review Board, and the requirement for written informed consent for computational analysis of the images was waived.

### Clinical data

Clinical and laboratory data, collected from electronic patient records, consisted of age, sex, primary tumour location, TNM8, Eastern Cooperative Oncology Group performance status, tobacco use, alcohol intake, and HPV status. Outcome data included PFS and 5-year PFS (locoregional recurrence or distant metastases, yes vs no). PFS was defined as the period between the diagnosis and the date of progression or last follow-up.

### CT data acquisition

All study patients underwent a CT scan within 28 days before treatment initiation. CT images were acquired at HCUV with two scanners and following acquisition and reconstruction protocols according to standard operating procedures for diagnostic imaging at this institution. All images were of comparable diagnostic quality, with intravenous contrast injection and 0.5 mm to 13 mm slice thickness. Any CT scan that had imaging artefacts in more than 50% of slices with primary tumour mass present was excluded. Further details on acquisition parameters are detailed in Supplementary Table S1.

CT axial images were exported from the picture archiving and communication system and stored in digital imaging and communication in medicine (DICOM) format for further radiomics feature extraction.

### Image segmentation

CT images in DICOM format were anonymised and uploaded to the Quibim Precision platform (v2.6 Quibim S.L.). An HCUV radiologist with over 20 years of expertise in head and neck cancer imaging reviewed the images to identify the primary tumour in the slice with the largest tumour area. Then, experienced radiographers manually segmented the entire primary tumour across all applicable series, creating a 3D volume of interest (VOI). This delineation was conducted on a contrast-enhanced CT scan, which reduces interobserver variability for HNSCC delineation [20]. Contrast media was preferred for its ability to provide richer pixel intensity variations and better characterise tissue heterogeneity in HNSCC through radiomics analysis.

### Radiomics analysis

No image quality harmonisation technique was applied given that all images were generated at a single institution. The image analysis and ML model pipeline is summarised in Fig. S1 (Electronic Supplementary Material). The entire methodology was implemented in Python 3.9. The quality of the study in terms of radiomic analysis was assessed by applying the METRICS form (Supplementary Fig. S2 included in the Supplementary Material).

#### Feature extraction

Radiomics features were obtained using the texture analysis module integrated into Quibim Precision, which complies with the Image Biomarker Standardisation Initiative [IBSI] [21]. Images were resampled to an isotropic voxel of 1 mm^3^ using a b-spline interpolation and normalised following the *z*-score strategy. A total of 108 3D radiomics features providing information on tissue heterogeneity and shape characteristics of the tumour (shape-based, first-order and second-order features) were extracted from each VOI and from the original image. For image discretisation, a fixed bin width of 25 was used.

#### Univariate analysis

Each radiomics feature was analysed for statistically significant distribution differences between the 5-year progression vs non-progression groups. The correlation between features showing differences was analysed. When two features showed a correlation higher than 0.9, the one with the most significant difference between progressors and non-progressors was kept.

#### Modelling

The three main models built were a clinical model (C1) based on the patient TNM stage, a comprehensive clinical model (C2) with TNM and significant clinical variables, and a radiomics-based model (C + R) constructed with both clinical and radiomics features to predict 5-year progression. Clinical features with more than 20% missing values were discarded. The remaining missing values were imputed using a k-nearest neighbours approach. KNNImputer, from scikit-learn, was used for data imputation with a number of neighbours set to 5. For model development and evaluation purposes, the dataset was randomly split into training-validation (80%) and test (20%), stratifying patients according to 5-year progression. A 5-fold cross-validation process was conducted in the training-validation set for hyperparameter tuning and to help detect model overfitting [22]. Correlations between radiomics features in the training dataset were analysed on each cross-validation fold. Next, during cross-validation, different solutions for feature standardisation, feature selection and ML models were tuned. Supplementary Table S2 lists the different evaluated and optimised options for each step. Scikit-learn (v.1.3.1) was used to implement all the previous processes. Apart from native scikit-learn models, scikit-survival (v.0.22.2) and extreme gradient boost (XGBoost) (v.2.0.3), were used for the models’ implementation. Furthermore, the best hyperparameters for each ML model were selected. The tuning process in cross-validation was performed using Optuna (v.3.5.0) to maximise the model’s performance in the validation dataset and was quantified with *c*-index for the PFS model and the area under the receiver operational curve (AUC) for the 5-year progression model. The evaluated hyperparameters are listed in Supplementary Tables S3 and S4.

#### Performance evaluation

To evaluate model performance on a new unseen dataset, the best configuration selected was re-trained over the whole training and validation dataset for final performance evaluation over the test set. For PFS prediction, the *c*-index, the *c*-index on inverse probability of censoring weights (IPCW), and dynamic AUC (dynAUC) metrics were used for the model’s evaluation. For the 5-year progression prediction, the evaluation metrics included sensitivity, specificity, accuracy, and AUC. All the metrics calculated were provided to evaluate the model’s overfitting and generalisation capabilities.

#### Explainability

To understand which selected features most strongly influenced model predictions and in which direction, SHAP values for each selected feature were extracted from the final model.

### Statistical analysis

Statistical analysis of continuous variables was conducted using Shapiro–Wilk’s test and Levene’s test for homoscedasticity. Student’s *t*-test compared normal distributions with homoscedasticity; Welch’s *t*-test was used for normal distributions without homoscedasticity, and the Wilcoxon rank sum test for non-normal distributions. Categorical variables were analysed using the exact Fischer test due to the small dataset size, with significance set at *p* < 0.05. Pearson’s coefficient assessed correlation in normal distributions and Spearman’s coefficient in non-normal distributions. Analyses were conducted using SciPy (v.1.9.3). PFS curves were compared by log-rank test.

## Results

### Patient characteristics

**Fig. 1:**
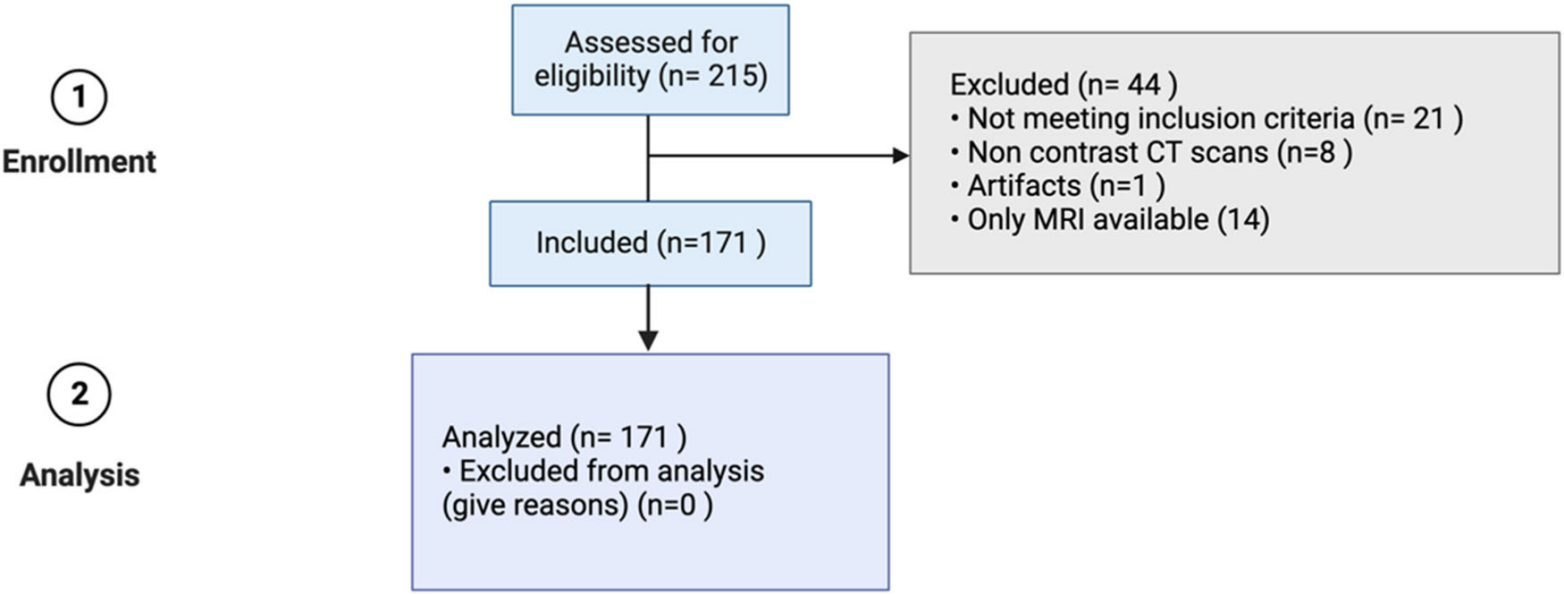
Flowchart of patient enrolment and inclusion/exclusion criteria

Data from a total of 215 patients (collected retrospectively from 63 patients and prospectively from 152) were considered for this study. There were no differences in the collection or processing of prospective and retrospective data. In total, eight patients were excluded from the analysis due to the absence of contrast CT scans, while one patient was excluded for image artefacts at the tumour region. Further exclusion criteria ruled out 14 patients for whom only magnetic resonance imaging images of the primary tumour were accessible, and 21 patients who failed to meet the predefined study inclusion criteria (Fig. 1). This left 171 eligible patients finally included in the study.

Patients’ demographic, clinical, and pathological characteristics are outlined in Table 1. Most study patients (*n* = 142, 83%) were male, the median age (range) at diagnosis was 62 (35–80) years, and the majority (*n* = 150, 88%) were smokers. The most frequent location of the primary tumour was the oral cavity (*n* = 63, 37%), followed by the larynx (*n* = 50, 30%). According to the TNM8, 94 (55%) patients were stage IVA and 36 (21%) were stage IVB. In total, 53% of patients (*n* = 91) experienced progression at 5 years.

**Table 1:** Patients’ demographic, clinical and pathological data

Variable	*N* = 171, *n* (%)	TRAIN/VAL *N* = 136, *n* (%)	TEST *N* = 35, *n* (%)	*p*
Age (years), median (range)	62 (35–80)	62 (35–80)	63 (50–80)	0.16
Sex				0.45
Male	142 (83)	111 (82)	31 (89)	
Female	29 (17)	25 (18)	4 (11)	
Tobacco use				0.54
Yes	150 (88)	120 (88)	30 (86)	
No	18 (10)	13 (10)	5 (14)	
Unknown	3 (2)	3 (2)	0 (0)	
Alcohol consumption				0.70
Yes	101 (59)	82 (60)	19 (54)	
No	66 (39)	51 (38)	15 (43)	
Unknown	4 (2)	3 (2)	1 (3)	
Primary tumour location				
Larynx and hypopharynx	68 (40)	54 (40)	14 (40)	1.00
Oropharynx	38 (22)	30 (22)	8 (23)	0.66
Oral cavity	63 (37)	51 (37)	12 (34)	0.70
Unknown primary of head and neck	2 (1)	1 (1)	1 (3)	
TNM stage				
II	4 (2)	4 (3)	0	0.58
III	37 (22)	33 (24)	4 (11)	0.11
IVA	94 (55)	70 (52)	24 (69)	0.09
IVB	36 (21)	29 (21)	7 (20)	1.00
HPV status				1.00
Positive	18 (10)	15 (11)	3 (9)	
Negative	87 (51)	69 (51)	18 (51)	
Not performed or not determined	66 (39)	52 (38)	14 (40)	
5-year progression (local or distant)				1.00
Yes	91 (53)	72 (53)	19 (54)	
No	80 (47)	64 (47)	16 (46)	
Median PFS (years (95% CI))	1.98 (0.42–0.58)	2.20 (0.40–0.58)	1.59 (0.29–0.64)	0.69

For model development and testing, 136 cases were used for training and validation while the remaining 35 cases were held for testing. No statistically significant differences were found between the two sets in any clinical variables or radiomics features.

#### Radiomics features

For each patient, 108 radiomics features were extracted. Statistically significant differences were observed in only three radiomics features across different scanners used for image acquisition: kurtosis (*p* = 0.007), range (*p* = 0.046), and grey level co-occurrence matrix Idmn (*p* = 0.049). Given the small number of features with differences and their relatively low statistical significance (*p* > 0.001), no further processing was performed.

### PFS model

To predict PFS, the R + C, and C1 and C2 models showed similar results, with IPCW of 0.67, *c*-index of 0.66, and dynAUC of 0.64 on the test set for the R + C model (Table 2). The ML model showing the best results was based on a random survival forest (RSF) after applying a min-max standardisation to radiomics features. A total of four radiomics features and one clinical variable were selected during cross-validation to build the final model. Specific hyperparameters are listed in Supplementary Table S5.

**Table 2:** Performance of the PFS model (RSF algorithm), using TNM8 staging (C1), TNM8 and clinical significant variables (C2) and clinical and radiomics features (R + C) as input to the model, overtraining, validation, and test subsets of data

	IPCW			*c*-Index			dynAUC		
	C1	C2	R + C	C1	C2	R + C	C1	C2	R + C
Train	0.61 (0.02)	0.61 (0.02)	0.78 (0.01)	0.60 (0.02)	0.61 (0.02)	0.78 (0.01)	0.65 (0.02)	0.57(0.02)	0.87 (0.01)
Validation	0.57 (0.10)	0.58 (0.05)	0.67 (0.09)	0.57 (0.10)	0.56 (0.10)	0.67 (0.09)	0.58 (0.14)	0.57 (0.10)	0.74 (0.10)
Test	0.66	0.66	0.67	0.66	0.66	0.66	0.66	0.65	0.64

**Fig. 2:**
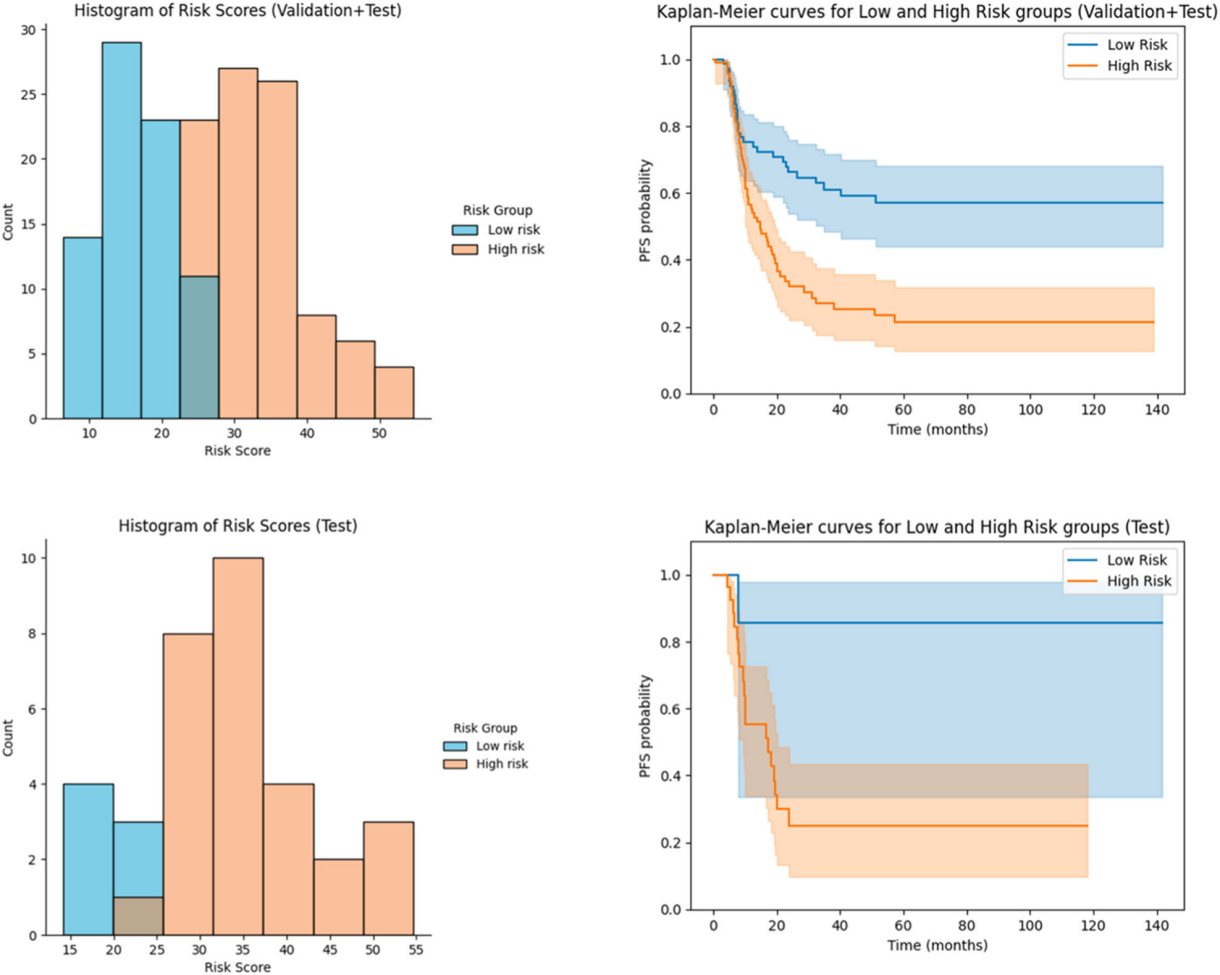
Histogram of risk scores (left) and Kaplan–Meier curves for low and high-risk patients according to R + C model PFS in both the validation and test dataset (upper row) and test dataset (bottom row)

The R *+* C model allowed patient stratification into low- and high-risk-progression according to the calculated cut-off of 24.37, through the Otsu method [22]. Kaplan–Meier survival curve analysis demonstrated significant statistical differences between the high-risk and low-risk cohorts in the overall cohort, training cohort, and test cohort (Log-rank *p* *<* 0.005, HR 1.23 95% confidence interval (95% CI: 0.60–2.54), as shown in Fig. 2.

### Five-year progression model

#### Univariate analysis

**Fig. 3:**
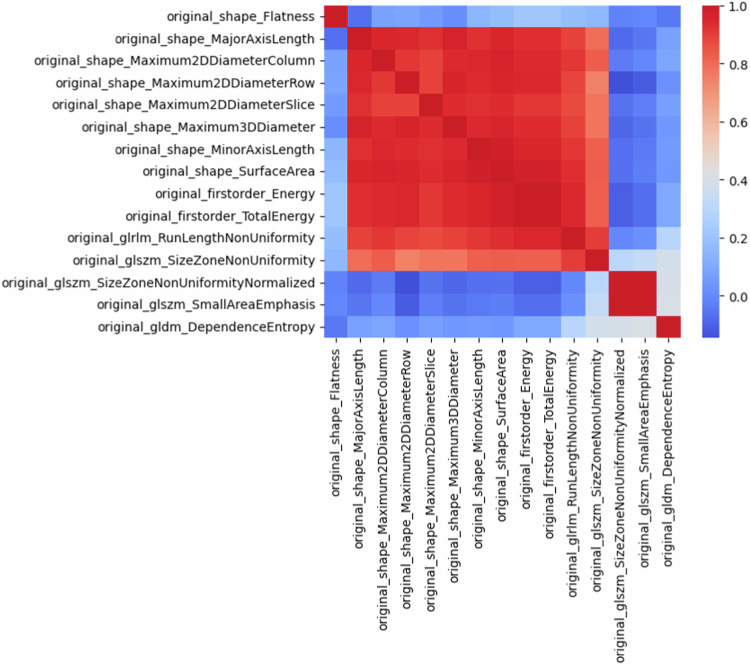
Correlation matrix (Spearman’s coefficient) between radiomics features showing statistically significant differences

Supplementary Table S6 lists all the radiomics features with statistically significant differences between 5-year progressors and non-progressors. A total of eight shape-based features, two first-order features and five second-order features showed differences. Correlation analysis (Fig. 3) revealed a high correlation (> 0.9) between most shape-based and first-order radiomics features. After selecting those with the most statistically significant differences between both groups, a total of two shape features (flatness and major axis length) and three second-order features (grey level size zone matrix (GLSZM) size zone non-uniformity, GLSZM small area emphasis (SAE) and grey level dependence matrix (GLDM) dependence entropy) were kept.

**Fig. 4:**
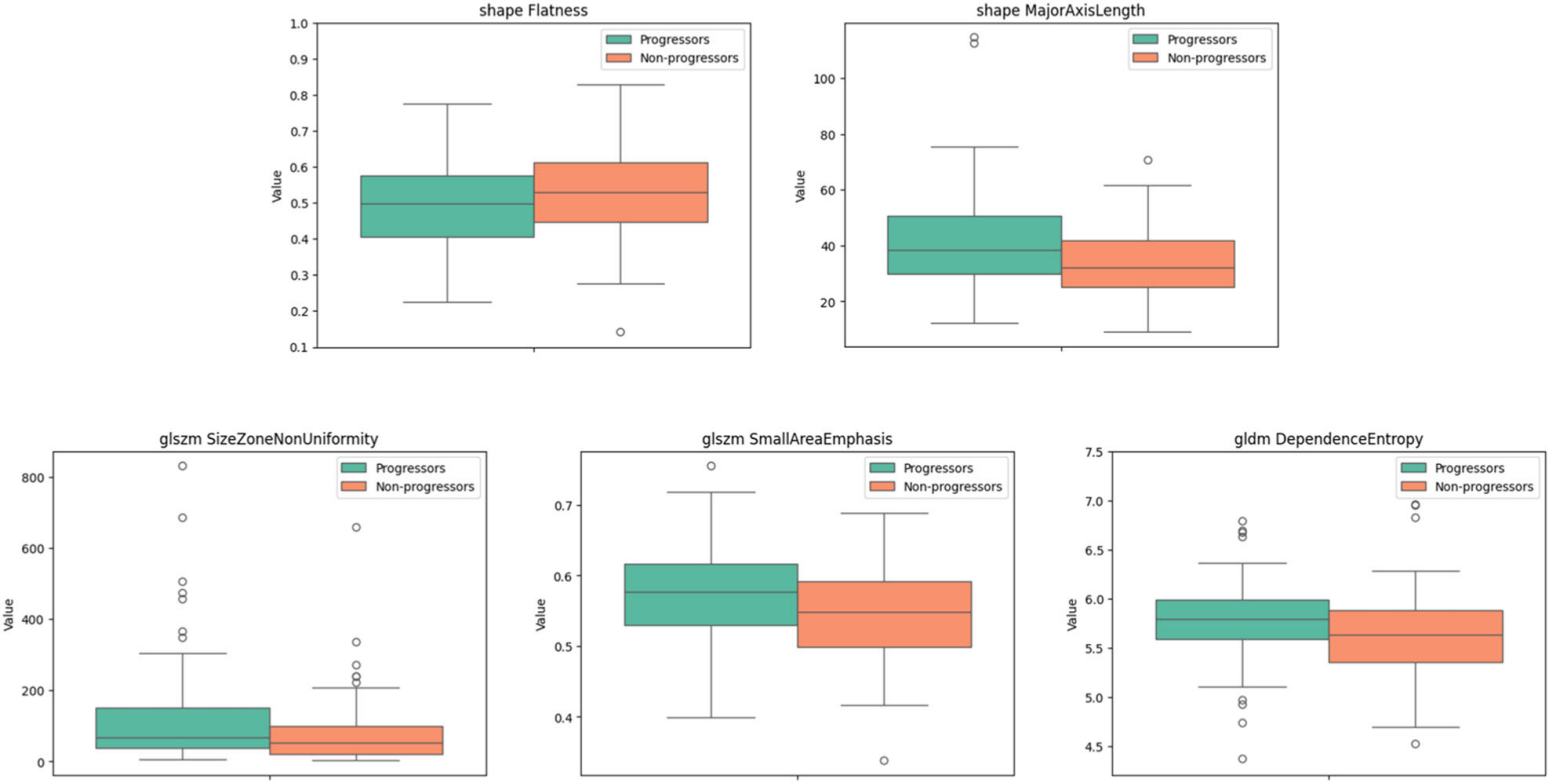
Boxplot distributions of some radiomics features showing statistically significant differences between 5-year progression and no-progression patients

Figure 4 shows the boxplot distributions for the previously selected features in 5-year progression and non-progression groups. The major axis length plot revealed that the largest diameter of the primary tumour was longer in progressors, while the second-order features revealed a more heterogeneous primary tumour in this same group.

#### Multivariate analysis

The results showed that the combined model (C + R) achieved higher AUC values compared to the models built with clinical information alone (C1 and C2), with AUCs of 0.74, 0.57, and 0.56, respectively. Additionally, a better balance between sensitivity and specificity was obtained in the C + R model, with a sensitivity of 0.53 and specificity of 0.81, compared to the C1 and C2 models. The performance of each model on training, validation (cross-validation results) and test subsets can be found in Table 3.

**Table 3:** Performance metrics obtained in 5-year progression prediction using TNM8 staging (C1), TNM8 and clinical significant variables (C2) and clinical and radiomics features (R + C) as input to the model, overtraining, validation and test subsets of data

	AUC			Sensitivity			Specificity			Accuracy		
	C1	C2	C + R	C1	C2	C + R	C1	C2	C + R	C1	C2	C + R
Training	0.58 (0.03)	0.62 (0.02)	0.81 (0.02)	0.81 (0.02)	0.71 (0.02)	0.68 (0.04)	0.36 (0.03)	0.75 (0.02)	0.80 (0.01)	0.60 (0.02)	0.62 (0.02)	0.74 (0.02)
Validation	0.58 (0.10)	0.62 (0.07)	0.75 (0.09)	0.81 (0.09)	0.71 (0.09)	0.67 (0.23)	0.36 (0.12)	0.75 (0.06)	0.78 (0.05)	0.60 (0.10)	0.62 (0.08)	0.72 (0.12)
Test	0.57	0.56	0.74	0.95	0.74	0.53	0.19	0.69	0.81	0.60	0.51	0.66

Mean and standard deviation values across the 5-fold cross-validation process were calculated for training and validation values

The ML model showing the best results was based on an XGBoost classifier after applying a standard scaling of the radiomics features. A total of 12 radiomics features and four clinical variables were selected during cross-validation to build the final model. Specific hyperparameters are listed in Supplementary Table S5. When comparing the selected features with those identified in the univariate analysis, three radiomics features were found to be significant in both analyses: flatness major axis length and SAE.

**Fig. 5:**
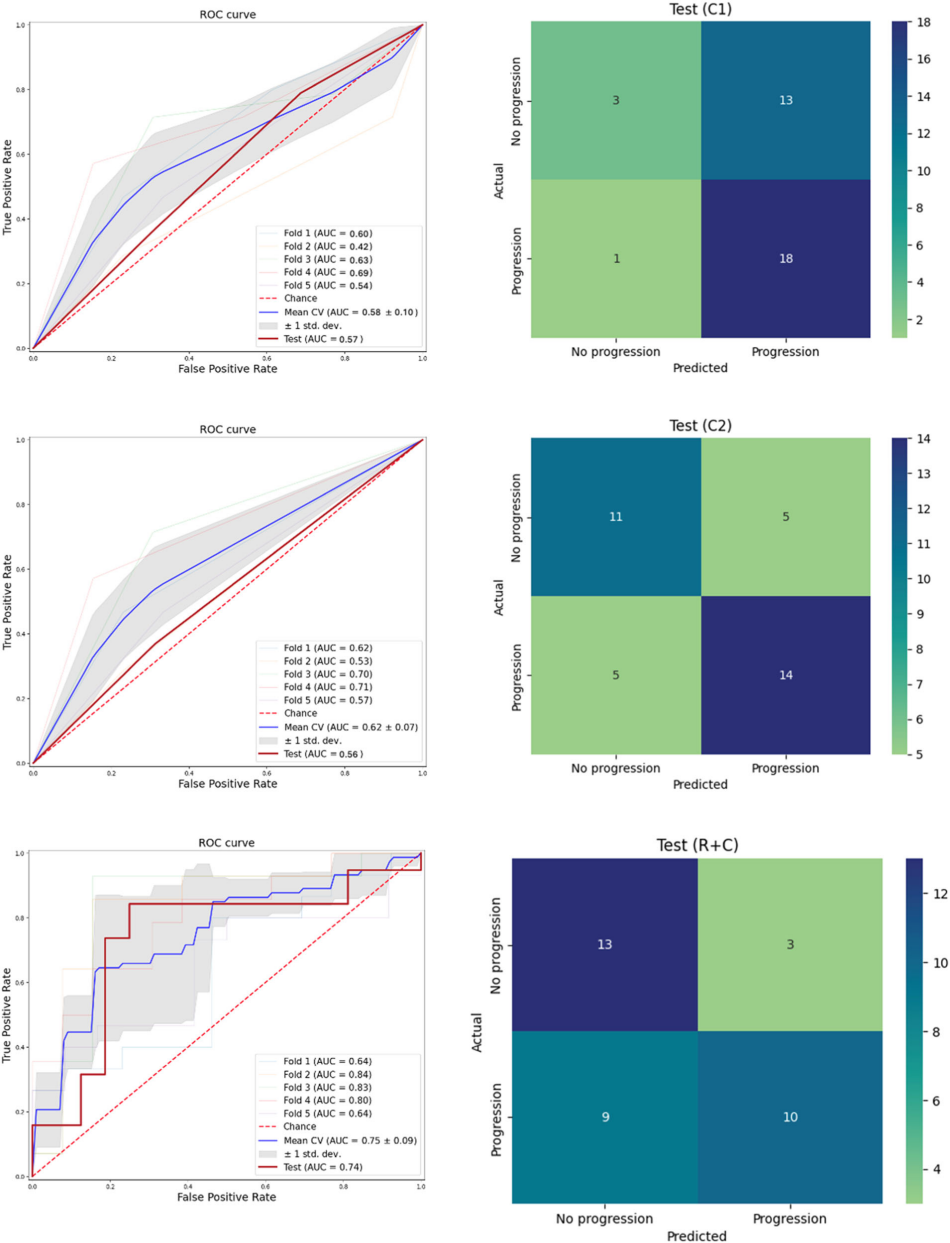
Left: Receiver operational curve on each fold of the cross-validation process and on the test set (dark red curve). Right: confusion matrix over the test dataset. Top row: C1 model. Middle row: C2 model. Bottom row: R + C model

Figure 5 shows the AUC of the C + R model on each fold of the cross-validation process and over the test dataset; the confusion matrix over the test set is also represented. The AUC over the test set was consistent with the one obtained during the cross-validation process, demonstrating the generalisation of the model to new unseen data. When evaluating the confusion matrix, 13 out of 16 patients without progression at 5 years were correctly classified, while 10 out of 19 progressors were properly classified.

### Interpretability

**Fig. 6:**
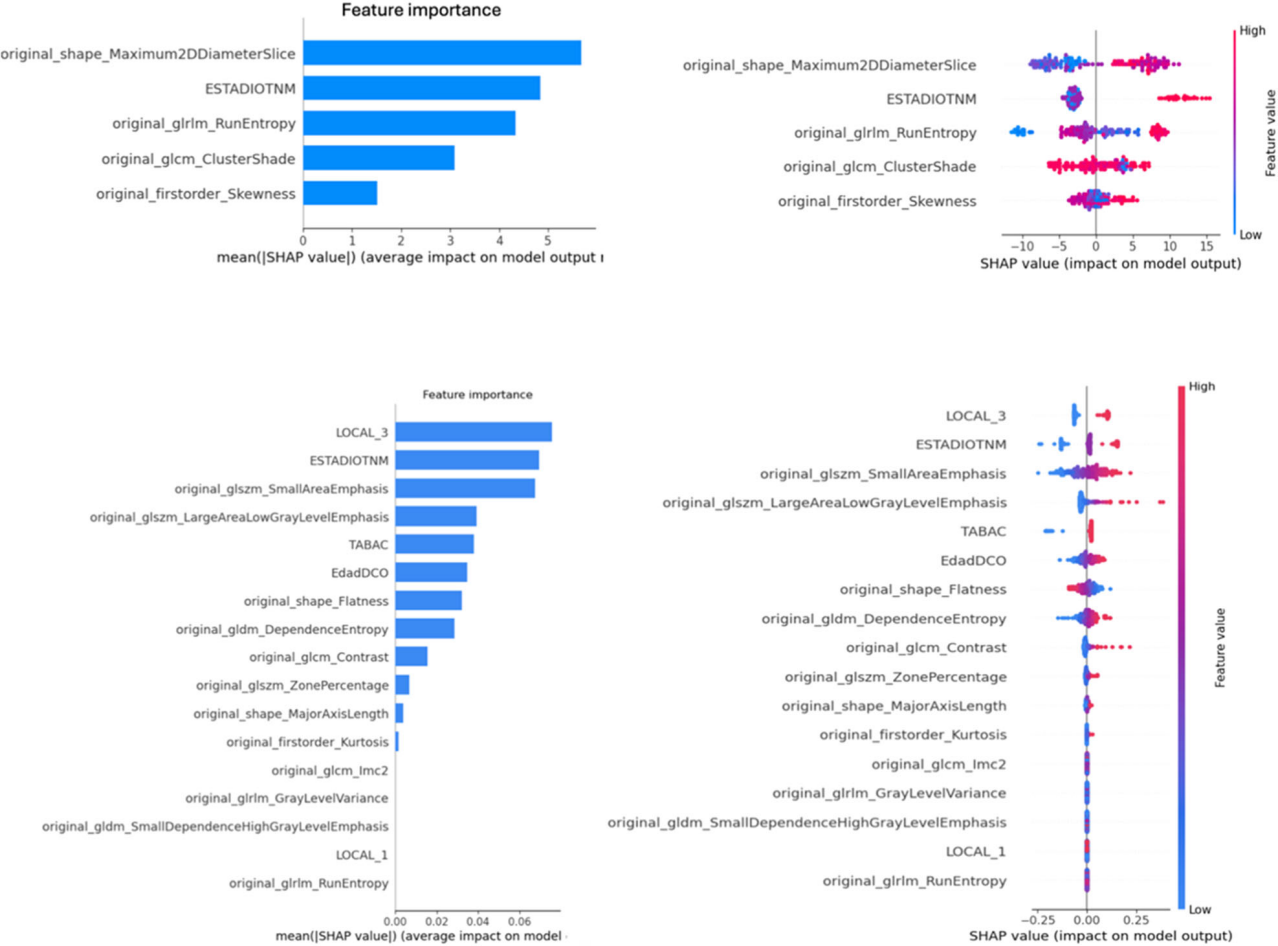
Left: feature importance of the selected features. Right: SHAP summary plots of the C + R model (test dataset), for each feature, dots to the right are related to a higher risk of progression, while they have a negative impact if they are positioned on the right; also, red dots are related to a higher value of the corresponding feature, while blue is related to a lower feature value. Top row: PFS model. Bottom row: 5-year progression model (LOCAL_3: primary tumour localisation in oral cavity; ESTADIOTNM: TNM stage; TABAC: tobacco consumption; EdadDCO: patient age at diagnosis; LOCAL_1: primary tumour localisation in larynx or hypopharynx)

Figure 6 presents the SHAP values for both PFS and 5-year progression models. For PFS prediction, the variable with the greatest impact was the maximum 2D diameter in the z-axis, highlighting the importance of the tumour extension. Patients with larger diameter values showed a higher risk of progression. Additionally, TNM staging had a significant influence on PFS prediction.

Regarding the 5-year progression model, the eight input features that most improved the model’s ability to stratify patients into risk groups (non-progression vs progression) were primary tumour localisation in the oral cavity, TNM stage, GLSZM SAE, GLSZM large area low grey lever emphasis, tobacco consumption, age at diagnosis, flatness and GLDM entropy. As shown in Fig. 6, tumour location in the oral cavity and elevated TNM were key risk factors for progression. Moreover, texture features such as emphasis and entropy, which reflect tumour heterogeneity, were significant radiomic predictors. As expected, tobacco consumption and age were also indicative of higher risk. As represented in Fig. 6, higher values of each feature were positively correlated with a higher risk of progression, except for the flatness shape feature which showed the opposite effect.

## Discussion

The variation in oncological outcomes among LAHNSCC patients after definitive chemoradiation (ChRT) highlights a need for improved risk stratification. This study aimed to develop a comprehensive prediction model by integrating clinical factors and radiomics features from baseline CT images, with the primary goal of estimating 5-year recurrence risk in non-surgical LAHNSCC patients undergoing radical ChRT. Emphasising clinical utility and explainability, the study employed the SHAP methodology to enhance understanding of ML-based models for treatment decision-making. This research represents the first application of SHAP in a tailored radiomics model for this patient population.

Only a handful of studies have been published on CT-based radiomics models in LAHNSCC with PFS as the primary objective [23–25], reporting AUC or CIs ranging around 0.72–0.74. Their main weaknesses lie in their small sample sizes (110–120 patients), exclusively retrospective design and lack of a validation cohort. Other challenges concern extrapolation, particularly when models are focused on specific anatomical locations such as the oropharynx or hypopharynx.

In our test set, the model utilising solely clinical data (C) showed an AUC of 0.57, while a notable improvement was observed upon integrating radiomics features (C + R), resulting in an AUC of 0.74. Particularly noteworthy is the substantially higher sensitivity achieved by the C model compared to the C + R model (0.95 vs 0.53). While the C model appears the most sensitive, this comes at the expense of specificity, which was markedly lower (0.19) than the enhanced specificity achievable with the inclusion of radiomics (0.81). These findings suggest that compared with using clinical data alone, the incorporation of radiomics might assist oncologists in more accurately identifying patients who are unlikely to experience relapse.

Although major axis length was a relevant shape-based radiomics feature presenting differences between progressor groups, it was not among those with higher weight in the model, highlighting the limitation of current tools to assess response criteria, such as response evaluation criteria in solid tumours 1.1, which are based only on tumour diameter changes. The most relevant radiomics features for the C + R model in order of importance were GLSZM SAE, GLSZM large area low grey level emphasis (LALGLE), flatness (shape) and GLDM entropy.

GLSZM analyses grey-level zones in an image. A grey-level zone is defined as the number of connected voxels that share the same grey-level intensity. The SAE is a measure of the distribution of small-size zones, with a greater value indicative of predominantly smaller-size zones and more fine textures, instead of large clusters of similar grey-level intensities. The LALGLE measures the proportion in the image of the joint distribution of larger-size zones with lower grey-level values. Flatness is a measurement of the spatial shape of the lesion, with values ranging between 1 (non-flat, sphere-like) and 0 (flat object). Finally, GLDM quantifies grey-level dependencies in an image. A grey-level dependency is defined as the number of connected voxels within a specific distance that is dependent on the centre voxel. Entropy represents uncertainty/randomness in the image values. The relevant radiomics features highlighted in the C + R model represent the presence of small same greyscale regions, large low greyscale regions, the flatness of the lesion and the degree of randomness in greyscale intensities. Except for flatness, the radiomics measurements pertinent to the C + R model captured characteristics of both small and large areas, as well as the randomness in their distribution. These features align with the heterogeneous nature of HNSCC.

A study by Keek et al [25] identified certain radiomic features overlapping with our study, such as GLSZM grey level and GLDM entropy. It is methodologically sound, prospectively validating a radiomic signature originally proposed by Parma [26] in a large cohort (*n* = 809). Their model accurately predicts OS despite risk stratification into three groups, differing in performance from the original study [26]. However, the study is limited by treatment heterogeneity and the use of clinical scales not commonly used in practice, potentially hindering its applicability.

Beyond its indisputable contribution to enhancing the interpretability of these models for clinicians, using the SHAP approach provides originality to our work. The only precedent for this approach is a recent study by a Scandinavian group focused solely on oropharyngeal cancer [26]. This large-scale study included a total population of 3284 patients, with 3164 in the training set and 120 in the temporary validation set. Impressively the study achieved an AUC of 0.93 in the validation cohort. However, it is important to note that the study allowed various treatment modalities, which could complicate definitive conclusions. Furthermore, it is noteworthy that despite being an integrated radiomic and clinical model, the most influential variables were exclusively clinical in nature.

The main limitation of our study is the small sample size, which can lead to overfitting in radiomics analyses. To mitigate this, we limited model signature sizes, through a feature selection process, to include only relevant features. Additionally, the limited number of events and the short observation period prevented the use of prospective data for model validation. Consequently, both datasets were pooled and randomly split into training and test sets. External prospective validation on a larger cohort is needed to ensure the accurate extrapolation and reproducibility of the results. Additionally, another limitation of the study is the inter- and intra-observer reproducibility, as the lesion labelling was performed solely by an experienced radiologist. However, to minimise variability and reduce segmentation errors, all segmentations were reviewed and refined in 3D by qualified imaging technicians. In cases of uncertainty regarding any labelling, the manual segmentation of the primary tumour was cross-checked by another imaging technician or radiologist to ensure the accuracy of the boundaries.

## Conclusion

A comprehensive model for LAHNSCC based on CT-radiomics features and including routine clinical variables shows remarkable accuracy in predicting PFS and stratifying risk groups, outperforming current clinical models. Prospective external validation is required to confirm these findings.

## Supplementary information

ELECTRONIC SUPPLEMENTARY MATERIAL

## Abbreviations

95% CI: 95% Confidence interval
AUC: Area under the curve
ChRT: Chemoradiation
CI: *c*-Index
CT: Computed tomography
DICOM: Digital imaging and communication in medicine
dynAUC: dynamic AUC
GLDM: Grey level dependance matrix
GLSZM: Grey level size zone matrix
HCUV: Hospital Clínico Universitario de Valencia
HPV: Human papillomavirus
IPCW: Inverse probability of censoring weights
LAHNSCC: Locally advanced head and neck cancer
LALGLE: Large area low grey level emphasis
ML: Machine learning
PFS: Progression-free survival
RSF: Random survival forest
SAE: Small area emphasis
SHAP: Shapley additive explanations
TNM8: Tumour-node-metastases eight edition
VOI: Volume of interest
XGBoost: Extreme gradient boost
