# Supplementary material for: CT-based clinical-radiomics model to predict progression and drive clinical applicability in locally advanced head and neck cancer
Source: Eur Radiol. 2024 Dec 20;35(7):4277–88. doi: 10.1007/s00330-024-11301-6 (PMC12165904; doi:10.1007/s00330-024-11301-6)
Supplement: Supplementary file 1 — ELECTRONIC SUPPLEMENTARY MATERIAL [file 330_2024_11301_MOESM1_ESM.pdf]

CT-based clinical-radiomics model to predict progression and drive clinical applicability in locally advanced head and neck cancer

ELECTRONIC SUPPLEMENTARY MATERIAL

Inclusion and exclusion criteria

The inclusion criteria were: 1) LAHNSCC confirmed by histopathology; 2) candidate for curative ChRT; 2) age > 18 years; 3) Eastern Cooperative Oncology Group Performance Status 0-1; 4) contrast-enhanced CT examination at baseline, and 5) at least one measurable lesion at baseline according to RECIST 1.1

The exclusion criteria were as follows: 1) Head and neck tumours with histology other than squamous cell carcinoma; 2) LAHNSCC patients for surgery only and/or adjuvant ChRT; 3) the absence of contrast-enhanced CT baseline images; 4) artifacts that influenced the evaluation on CT and 5) the lack of sufficient clinical data.

Supplementary Figure S1

Figure S1. Radiomics workflow.

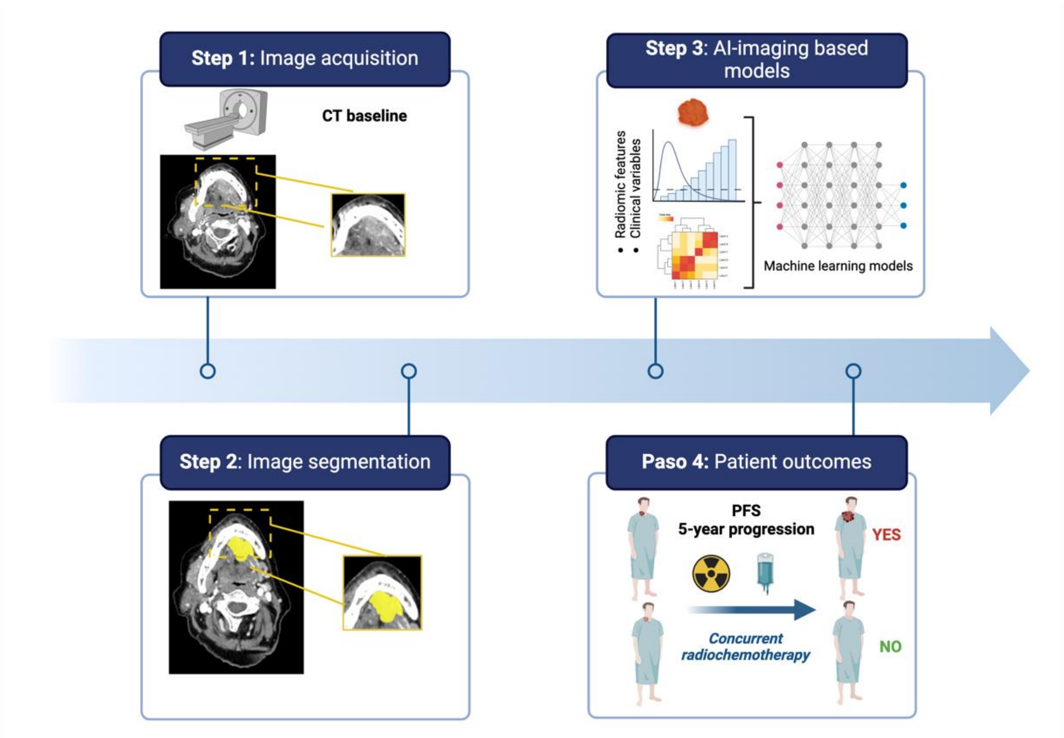

Supplementary Figure S2

Figure S2. METRICS form to assess the quality of the study.

15/10/2024, 17:54 METRICS

METRICS Tool v1.0

Please fill out all conditions first for relevant sections and then all active items to calculate METRICS score.

Please note that default option is "No".

? Stands for explanation of items and conditions.

C Stands for conditional items or sections.

Items/Conditions	Definitions	Weights	Options
**Study Design**			
Item#1	? Adherence to radiomics and/or machine learning-specific checklists or guidelines	0.0368	◉ Yes ○ No
Item#2	? Eligibility criteria that describe a representative study population	0.0735	◉ Yes ○ No
Item#3	? High-quality reference standard with a clear definition	0.0919	◉ Yes ○ No
**Imaging Data**			
Item#4	? Multi-center	0.0438	○ Yes ◉ No
Item#5	? Clinical translatability of the imaging data source for radiomics analysis	0.0292	◉ Yes ○ No
Item#6	? Imaging protocol with acquisition parameters	0.0438	◉ Yes ○ No
Item#7	? The interval between imaging used and reference standard	0.0292	◉ Yes ○ No
**Segmentation** C			
Condition#1	? Does the study include segmentation?		◉ Yes ○ No
Condition#2	? Does the study include fully automated segmentation?		○ Yes ◉ No
Item#8	? Transparent description of segmentation methodology	0.0337	◉ Yes ○ No
Item#9	? Formal evaluation of fully automated segmentation C	0.0225	○ Yes ○ No
Item#10	? Test set segmentation masks produced by a single reader or automated tool	0.0112	◉ Yes ○ No
**Image Processing and Feature Extraction**			
Condition#3	? Does the study include hand-crafted feature extraction?		◉ Yes ○ No
Item#11	? Appropriate use of image preprocessing techniques with transparent description	0.0622	◉ Yes ○ No
Item#12	? Use of standardized feature extraction software C	0.0311	◉ Yes ○ No
Item#13	? Transparent reporting of feature extraction parameters, otherwise providing a default configuration statement	0.0415	◉ Yes ○ No
**Feature Processing**			
Condition#4	? Does the study include tabular data?		◉ Yes ○ No
Condition#5	? Does the study include end-to-end deep learning?		○ Yes ◉ No
Item#14	? Removal of non-robust features C	0.0200	○ Yes ◉ No
Item#15	? Removal of redundant features C	0.0200	◉ Yes ○ No
Item#16	? Appropriateness of dimensionality compared to data size C	0.0300	◉ Yes ○ No
Item#17	? Robustness assessment of end-to-end deep learning pipelines C	0.0200	○ Yes ○ No
**Preparation for Modeling**			
Item#18	? Proper data partitioning process	0.0599	◉ Yes ○ No
Item#19	? Handling of confounding factors	0.0300	◉ Yes ○ No
**Metrics and Comparison**			
Item#20	? Use of appropriate performance evaluation metrics for task	0.0352	◉ Yes ○ No
Item#21	? Consideration of uncertainty	0.0234	◉ Yes ○ No
Item#22	? Calibration assessment	0.0176	○ Yes ◉ No
Item#23	? Use of uni-parametric imaging or proof of its inferiority	0.0117	◉ Yes ○ No
Item#24	? Comparison with a non-radiomic approach or proof of added clinical value	0.0293	◉ Yes ○ No
Item#25	? Comparison with simple or classical statistical models	0.0176	○ Yes ◉ No
**Testing**			
Item#26	? Internal testing	0.0375	◉ Yes ○ No
Item#27	? External testing	0.0749	○ Yes ◉ No
**Open Science**			
Item#28	? Data availability	0.0075	○ Yes ◉ No
Item#29	? Code availability	0.0075	○ Yes ◉ No
Item#30	? Model availability	0.0075	○ Yes ◉ No
		**Total METRICS score:**	79.5%
		? **Quality category:**	Good
		? **Publication ID:**	

If you publish any work which uses this tool, please cite the following publication:

Kocak B, Akinci D'Antonoli T, Mercaldo N, et al. METhodological RadiomICs Score (METRICS): a quality scoring tool for radiomics research endorsed by EuSoMII. Insights Imaging. 2024;15(1):8. Published 2024 Jan 17. doi:10.1186/s13244-023-01572-w

Supplementary Table S1

Table S1. CT data acquisition parameters

	GE Revolution EVO	TOSHIBA Aquilion
**Tube voltage (kVp)**	120	120
**Tube current (mA)**	Dynamic	Dynamic
**Rotation time (s)**	0.8	0.5
**Beam collimation (mm)**	64 x 0.625	80 x 0.5
**Acquisition mode**	Helical	Helical
**Section thickness (mm)**	13	5
**Section interval (mm)**	13	5
**Kernel**	Standard	FC08
**Reconstruction algorithm**	ASIR-V	AIDR 3D
**Matrix**	512 x 512	512 x 512
**Field of view (mm x mm)**	302 x 302	242 x 242

Supplementary Table S2

Table S2. Feature selection, feature standardization and machine learning model configurations evaluated and tuned during cross-validation in both PFS and 5-year progression models.

	PFS	5-year progression
Feature selection	PCA, KPCA, MRMR, univariate cox, variable importance according to RSF	Statistical importance, PCA, KPCA, variable importance according to RF or according to GB.
Feature standardization	Min-max, standard, robust	Min-max, standard, robust
ML model	RSF, Cox PH, ElasticNet Cox, EST, GB survival analysis	RF, SVC, LR, GB, XGBoost.
Abbreviations: PCA (Principal Component Analysis), KPCA (Kernel Principal Component Analysis), MRMR (Minimum Redundancy Maximum Relevance), RSF (Random Survival Forest), PH (Proportional Hazards), EST (Extra Survival Trees), GB (Gradient Boosting). RF (Random Forest), SVC (Support Vector Classifier), LR (Logistic Regression).		

Supplementary Table S3

Table S3. Hyperparameters evaluated and tunned during cross-validation for each PFS machine learning model.

ML model	Hyperparameter	Value type	Value range
**RSF**	n_estimators	Integer	50 – 500
	max_depth	Integer	2 – 64
	min_samples_split	Integer	2 – 11
	min_samples_leaf	Integer	2 – 11
	max_features	Categorical	sqrt, log2
**Cox PH**	alpha	Logarithmic uniform distribution	0.001 – 1
	ties	Categorical	breslow, Efron
**ElasticNet Cox**	n_alphas	Integer	50 – 200
	l1_ratio	Float	0.1 – 0.9
	alpha_min_ratio	Float	10^{-5} – 0.1
**EST**	n_estimators	Integer	50 – 500
	max_depth	Integer	2 – 64
	min_samples_split	Integer	2 – 11
	min_samples_leaf	Integer	2 – 11
	max_features	Categorical	sqrt, log2
**GB survival analysis**	learning_rate	Float	0.001 – 0.9
	subsample	Discrete uniform distribution	0.2 – 1.0
	n_estimators	Integer	50 – 500
	max_depth	Integer	2 – 64
	min_samples_split	Integer	2 – 11
	min_samples_leaf	Integer	2 – 11
	max_features	Categorical	sqrt, log2

Supplementary Table S4

Table S4. Hyperparameters evaluated and tunned during cross-validation for each 5-years progression machine learning model.

ML model	Hyperparameter	Value type	Value range
RF	criterion	Categorical	entropy, gini
	max_features	Categorical	sqrt, log2
	min_samples_leaf	Integer	1 - 11
	max_depth	Integer	1 - 128
SVC	kernel	Categorical	(rbf, sigmoid)*, (linear, poly, rbf, sigmoid)**
	C	Logarithmic uniform distribution	1 - 100
	degree	Integer	1 - 5
	gamma	Float	0.01 - 10
LR	penalty	Categorical	l1, l2, elasticnet
	C	Logarithmic uniform distribution	0.1 - 10
	solver	Categorical	saga if penalty is elasticnet; newton-cg, lbfgs, sag, saga, liblinear otherwise
	l1_ratio	Float	0 - 1 if penalty if elasticnet
GB	n_stimators	Integer	100 - 2500
	learning_rate	Logarithmic uniform distribution	0.001 - 0.05
	criterion	Categorical	frieadman_mse, squared_error
	loss	Categorical	deviance, exponential
	subsample	Discrete uniform distribution	0.2 - 1.0
XGBoost	n_stimators	Integer	100 - 2500
	learning_rate	Float	0.001 – 0.05
	booster	Categorical	gbtree, gblinear, dart
	subsample	Discrete uniform distribution	0.2 – 1.0
	reg_alpha	Float	10^{-8} – 1
	reg_lambda	Float	10^{-8} – 1
	colsample_bytree	Float	0.2 – 1

Supplementary Table S5

Table S5. Radiomics features showing statistically significant differences (p-value<0.5) between 5-year progressors and non-progressors. With asterisk, those features selected after correlation analysis are highlighted.

Radiomics feature	Type	p-value
Flatness	Shape	0.028*
Major axis length	Shape	0.006*
Maximum 2D diameter column	Shape	0.013
Maximum 2D diameter row	Shape	0.042
Maximum 2D diameter slice	Shape	0.02
Maximum 3D diameter	Shape	0.005
Minor axis length	Shape	0.047
Surface area	Shape	0.043
Energy	First order	0.043
Total energy	First order	0.043
GLRLM Run length non-uniformity	Second order	0.033
GLSZM Size zone non-uniformity	Second order	0.020*
GLSZM Size zone non-uniformity normalized	Second order	0.026
GLSZM Small area emphasis	Second order	0.016*
GLDM Dependence entropy	Second order	0.037*
Abbreviations: GLRLM (Gray-Level Run Length Matrix), GLSZM (Gray-Level Size Zone Matrix), GLDM (Gray-Level Dependence Matrix		

Supplementary Table S6

Table S6. Final hyperparameters of the best model after cross-validation for both PFS and 5-year progression models.

PFS model		
	**Hyperparameter**	**Value**
**EST**	n_estimators	65
	max_depth	15
	min_samples_split	4
	min_samples_leaf	11
	max_features	sqrt
**5-year progression**		
**XGBoost**	n_stimators	1851
	learning_rate	0.025
	booster	gblinear
	subsample	0.6
	reg_alpha	0.015
	reg_lambda	$1.044 \cdot 10^{-8}$
	colsample_bytree	0.810
